# Atrophic Rhinitis

**Published:** 1904-01-16

**Authors:** 


### ATROPHIC RHINITIS.

Ozæna is a malady so distressing both to the patient and his associates that any suggestions with regard to its better treatment are welcome. Mr. Lambert Lack,[1] in discussing the condition, describes a method which in his hands appears to have met with a large measure of success in combating the unpleasant symptoms. His plan is based upon the pathology of the condition. He defines ozæna as a disease of the nose associated with an intensely fœtid muco-purulent discharge in the form of crusts, with undue width of the nasal fossæ, and with changes of an atrophic nature in the nasal mucous membrane. The disease almost always commences under 12 years of age, but is at first evidenced only by a purulent discharge, which is usually neglected. It appears that portions of the mucous membrane of the nose which are normally covered by ciliated epithelium come to have a lining of squamous epithelium. Consequently any secretion which forms, instead of being carried away to the throat by ciliary action, becomes desiccated in the nose. Moreover, as the nasal cavities are abnormally wide, the secretion cannot be removed by blowing the nose; the secretion dries and forms the crusts which are a feature of atrophic rhinitis. The discharge is at first fluid and non-fœtid. The fœtor forms subsequently and is due to decomposition of the crusts. Thus the bad odour and the crusts are secondary phenomena. Bearing these facts in mind the important point to be aimed at in treating a case is to cleanse the nose thoroughly and to keep it clean. The nose must be carefully syringed. A solution made by adding a teaspoonful of salt to a pint of water is as good as anything. It is not a question of antiseptics but of mechanical removal of the discharge. Having washed the nose out it is well to inspect it and to detach or to syringe away any crusts that remain. After this has been done the most important step remains, namely, to pack the nose with strips of cyanide gauze. A strip an inch wide and from 12 to 20 inches long should be inserted, an operation which the patient can be taught to carry out himself. This packing prevents the entrance of air into the nose, and so obviates the drying and decomposition of the discharge. The result is that the discharge remains fluid, the nose no longer contains tenacious crusts, and can easily be cleansed by syringing. The nose should be syringed twice daily, and the use of the gauze packing should not be discontinued. The patients soon learn to tolerate any discomfort which may be felt at first, and are glad to adopt a plan of treatment by which the main symptoms of their complaint can be entirely removed.

[1] Clinical Journal, Dec. 23, 1903.

